# Contraceptive acceptability and associated factors among young women (15–24) living with HIV/AIDS: a hospital-based study in Kampala, Uganda

**DOI:** 10.4314/ahs.v22i1.4

**Published:** 2022-03

**Authors:** Muzeyi Wani, Janet Nakigudde, Hildah Tendo Nansikombi, Philip Orishaba, Dennis Kalibbala, Joan N Kalyango, Steven M Kiwuwa

**Affiliations:** 1 Clinical Epidemiology Unit, School of Medicine, College of Health Sciences, Makerere University, Kampala, Uganda; 2 Department of psychiatry, School of medicine, College of Health Sciences, Makerere University, Kampala, Uganda; 3 Child Health Development Center, College of Health Sciences, Makerere University, Kampala, Uganda

**Keywords:** Contraceptive acceptability, young women, HIV/AIDS, Kampala, Uganda

## Abstract

**Introduction:**

In Uganda, over 43% of all pregnancies among young women (15–24 years) living with HIV are either unwanted or mistimed. Unintended pregnancies account for 21.3% of neonatal HIV infections. The objective was to determine acceptability of contraceptives and associated factors among young women living with HIV attending HIV clinics in Kampala.

**Methods:**

Between February and May 2019, 450 young women attending public HIV clinics (Kisenyi HC IV, Kiswa HC III and Komamboga HC III) in Kampala were systematically enrolled in a cross sectional study and interviewed using structured questionnaires. We used modified Poisson regression to determine the factors associated with acceptability of contraceptive. Data were analyzed using STATA 13.0. Statistical significance was determined at a P values < 0.05.

**Results:**

Contraceptive acceptability was 40.7% (95% CI: 27.6%–53.6%). Older age group (20–24 years) (aPR; 2.42, 95%CI; 1.06–5.52, P = 0.035), age at sex debut ≥ 18 years (aPR;1.25,95%CI; 1.13–1.38, P<0.001), having friend on contraceptives (aPR; 1.90, 95%CI; 1.10 – 3.26; P =0.021) and being married (aPR; 1.20, 95%CI; 1.09 – 1.32, P<0.001) were significantly associated with acceptability of contraceptives.

**Conclusion:**

There is a low acceptability for contraceptives. Younger age group who are not yet married need to be targeted.

## Introduction

Sub-Saharan Africa (SSA) accounts for 70% of the global human immunodeficiency virus (HIV) burden. Every week, around 7000 young women (15–24 years) become infected with HIV in SSA. Young women are also twice as likely to be living with HIV as men of the same age^1^. The prevalence of HIV among young women in Uganda is four times that of young men1 and young women have the highest rate of unwanted pregnancy at 43.9% in Uganda^2^. A study on sexual risk related behaviour among youth 15–24 years living with HIV (LHIV) in central Uganda reported that 45% were sexually active, of these 57% did not use any form of contraception despite wanting to delay pregnancy and 30% had more than one sexual partner in the last six months^3^. This high risk sexual behaviour together with low usage of contraceptives puts young HIV positive women at risk of unplanned pregnancy^4^. A survey on uptake of family planning services in Uganda reported that 43% of pregnancies among HIV positive women were either mistimed or unintended^5^ and unintended pregnancies accounted for 21.3% of neonatal HIV infections^6^. Although the Uganda national HIV treatment guidelines recommend integration of family planning services into routine HIV care^7^, uptake among young HIV positive women is still low. The study set out to determine acceptability of contraceptives and its associated factors among young women LHIV in Kampala city HIV clinics. The findings of the study could help reduce unwanted pregnancies among young women LHIV.

## Methods

### Study design and setting

This was a cross sectional study. The study was conducted at three Kampala capital city Authority (KCCA) health facilities namely Kisenyi Health centre (HC) IV, Kiswa and Komamboga HC III. All facilities offered a wide range of health care services in addition to HIV care and family planning services. All services offered were free of charge. Kisenyi HC IV had a separate HIV/AIDS clinic for adolescents and young. On average, the weekly attendance by the study participants at Kisenyi HC IV was 80 participants and 60 for Komamboga and Kiswa HCs. These facilities were purposively selected out of 10 HCs in the city due to their busy HIV clinics. The HIV clinic in these facilities runs Monday to Thursday, 8am to 5 pm.

### Study population

The study involved 15–24-year-old women LHIV that attended the three KCCA HIV clinics between February and May 2019 and had consented for the study.

### Ethics and consent

Permission to conduct the study was obtained from the Clinical Epidemiology Unit, MakCHS; institutional approval was obtained from the Makerere University School of Medicine Research thics Committee (SOMREC). Approval was granted ref no. REC 2019-032. Administrative permission was obtained from the directorate of public health KCCA and permission to access study participants was granted by facility in charges. All participants were requested to consent before participating in the study. Participants younger than 18 years were treated as emancipated minors. Confidentiality was ensured by using unique identifiers for participants.

### Sample size

Kish Leslie 1965 was used to calculate sample size as follows, Using a contraceptive acceptability of 52% 2 and precision of 0.05, assuming a 95% level of confidence, we obtained a sample size of 385 participants and a final sample size of 443 participants when adjusted for 15% non-responsiveness.

### Sampling

Participants were enrolled systematically. On each study day, every 5th eligible participant at the HIV clinic during the study period was selected. Each facility contributed a proportionate sample to the total sample size based on the total number of clients from each facility as a proportion of the total number of all clients in the three facilities. We, therefore recruited 192 participants from Kisenyi HC IV, 142 participants from Kiswa HC III and 116 participants from Komamboga HC III.

### Data collection and management

Between February and May 2019, 450 participants were enrolled. Data from each facility was collected using an interviewer administered questionnaire. The whole questionnaire was translated into Luganda, a common language besides English in Kampala. One HIV clinic nurse from each facility was trained on study procedures and recruited as a research assistant. The research assistants administered informed consent and then collected data using a structured questionnaire. The data was coded and then entered using a computer software (EPI data).

### Analysis

Data on socio-demographic, clinical and reproductive characteristics were summarized using frequencies and proportions and presented in tables. Acceptability of contraceptives was determined on the basis of the respondent's response to the question “How much do you like the idea of contraceptives for yourself?” This question was previously used by Whitaker et al in studies of an educational intervention regarding IUDs10. Response options were 1(Very acceptable), 2 (Acceptable), 3 (Not sure),4 (Unacceptable) and 5 (Very unacceptable). A binary variable recoding responses 1–2 as acceptable and 3–5 unacceptability was created. To assess associations between the independent variables and acceptability, we used modified Poisson regression with robust standard errors because acceptability was not rare outcome in this study (>25%). Independent variables with a p-value less than 0.2 at bivariate were considered for multivariate analysis. Associations with p-value < 0.05 at multivariate were considered statistically significant. Significant variables were checked for interaction and dropped variables checked for confounding. The results of the analysis are presented in tables 1 to 4. STATA 13.0 was used for the analysis.

**Table 1:** Social demographic characteristics of 450 young HIV positive women in KCCA HIV clinics

Variable	Frequency (n=450)	Percent (%)
**Age**		
<20	189	42.0
>=20	261	58.0
**Education**		
None	49	10.9
Primary	102	22.7
Secondary	221	49.1
Tertiary	78	17.3
**Occupation**		
Student	189	42
Salaried Job	78	17.3
Self employed	67	14.9
Unemployed	116	25.8
**Religion**		
Catholic	178	39.6
Protestant	111	24.7
Muslim	111	24.7
Other*	50	11.1
**Marital status**		
Single	286	63.6
Married	91	20.2
Cohabiting	58	12.9
Divorced	15	3.3
**Stay with parents**		
NO	225	50
Yes	225	50

Others*: 35 Pentecostal/450, 15/450 Jehovah's witness.

**Table 2:** Clinical and reproductive characteristics of 450 young HIV positive women in KCCA HIV clinics

Variable	Frequency (n=450)	Percent (%)
**Viral load suppression**		
Suppressed	376	83.6
Not Suppressed	74	16.4
**Ever had sex**		
No	139	30.9
Yes	311	69.1
**Age at sex debut**		
<18	167	53.7
>=18	144	46.3
**Have regular sex partner**		
No	144	36.7
Yes	197	63.3
**Number of sex partner**		
one	214	68.8
Multiple	97	31.2
**Ever had an abortion**		
No	255	81.9
Yes	56	18.1
**Parity**		
Nulliparous	26	8.4
para 1–2	265	85.2
>para 2	20	6.4
**Talk to partner about contraceptives**		
No	136	43.7
Yes	175	56.3
**Friends use contraceptives**		
No	144	32.0
Yes	306	68.0
**Contraceptive knowledge**		
No	226	72.7
Yes	85	27.3

**Table 3a:** Bivariate analysis of factors associated with acceptability of contraceptives

Variable	Acceptability of contraceptives		Crude PR	95% CI	P value
	Yes (n=183)	No (n=267)			
**Age**					
<20	33(17.5)	156(82.5)	1.00		
>=20	150(57.5)	111(42.5)	3.29	3.11–3.48	**<0.001**
**Viral load suppression**					
Suppressed	155(41.2)	221(58.8)	1.00		
Not Suppressed	28(37.8)	46(62.2)	1.09	0.82–1.45	0.558
**Ever had sex**					
No	27(19.4)	112(80.6)	1.00		
Yes	156(50.2)	155(49.8)	2.58	1.38–4.84	**0.003**
**Marital status**					
Single	87(30.4)	199(69.6)	1.00		
Married	58(63.7)	33(36.3)	2.10	1.87–2.34	**<0.001**
Cohabiting	31(53.5)	27(46.5)	1.76	1.12–2.75	**0.014**
Divorced	7(46.7)	8(53.3)	1.53	1.22–1.92	**<0.001**
**Stay with parents**					
NO	122(54.2)	103(45.8)	1.00		
Yes	61(27.1)	164(72.9)	0.50	0.35–0.70	**<0.001**

**Table 3b:** Bivariate analysis of factors associated with acceptability of contraceptives

Variable	Contraceptive acceptability		Crude PR	95% CI	P value
	Yes (n=156)	No (n=155)			
**Age at sex debut(n=311)**					
<18	64(38.3)	103(61.7)	1.00		
>=18	92(63.9)	52(36.1)	1.67	1.34–2.07	<0.001
**Have regular sex partner(n=311)**					
No	50(46.9)	64(56.1)	1.00		
Yes	106(53.8)	91(46.2)	1.23	1.05–1.43	**0.009**
**Number of sex partners(n=311)**					
one	102(47.7)	112(52.3)	1.00		
Multiple	54(55.7)	43(44.3)	1.17	0.87–1.57	0.308
**Ever had an abortion(n=311)**					
No	128(50.2)	127(49.8)	1.00		
Yes	28(50.0)	28(50.0)	0.99	0.81–1.21	**0.969**
**Parity(n=311)**					
Nulliparous	12(46.2)	14(53.8)	1.00		
para 1–2	131(49.4)	134(50.6)	1.07	0.71–1.63	0.747
>para 2	13(65.0)	7(35.0)	1.41	1.04–1.90	**0.025**
**Friends use contraceptives**					
No	29(20.1)	115(79.9)	1.00		
Yes	154(50.3)	152(49.7)	2.45	1.16–5.38	**0.019**
**Contraceptive knowledge**					
No	103(45.6)	123(54.4)	1.00		
Yes	53(62.4)	32(37.6)	1.37	1.11–1.69	**0.003**

**Table 4:** Multivariate analysis of factors associated with acceptability of contraceptives

Variable	Crude PR	Adjusted PR	95% CI	P value
**Age**				
<20	1.00	1.00		
>=20	3.29	2.42	1.06 – 5.52	0.035
**Age at sex debut**				
<18	1.00	1.00		
>=18	1.67	1.25	1.13 – 1.38	<0.001
**Friends use contraceptives**				
No	1.00	1.00		
Yes	2.45	1.90	1.10 – 3.26	0.021
**Marital status**				
Single	1.00			
Married	2.10	1.20	1.09 – 1.32	<0.001
Cohabiting	1.76	0.99	0.71 – 1.39	0.969
Divorced	1.53	0.91	0.84 – 0.99	0.029

## Results

### Description of study population

A total of 450 participants were interviewed between February and May 2019. Fifty eight percent of the respondents were over 19 years old. Majority of the respondents (49.1%) had secondary level education, 63.6% were single and 30.9% had never had sexual intercourse (Tables 1 & 2).

### Acceptability of contraceptives and its associated factors

Acceptability of contraceptives was 40.7% with 95% confidence interval (CI): 27.6–53.6%). Tables 3a, 3b and 4 show the bivariate and multivariate analysis of factors associated with acceptability of contraceptives: age, age at sex debut, friend uses contraceptives and marital status (married and divorced) were significantly associated with contraceptive acceptability.

## Discussion

Contraceptives acceptability was low. There is generally a low acceptability of contraceptives among young women^12^. This highlights a lack of awareness about contraceptives in this age group. Age was found to be significantly associated with acceptability of contraceptives where participants older than 20 years were 2.42 times more likely to use contraceptives compared to those younger. A study on knowledge and use of birth control methods by young women^13^ also reported being under the age of 15 to be associated with no use of contraceptives. This could be because those younger have limited knowledge of contraceptives. There was a gap in comparative literature on the how age is associated with acceptability of contraceptives among age HIV positive women. Age at first sex was significantly associated with contraceptive acceptability. A study on early age at first sex and subsequent contraceptive use gap reported that girls younger than 15 years at the time of first sex were likely to have a gap in contraceptive use^14^. This is in agreement with the findings of our study. Marital status was significant factor associated with acceptability of contraceptives in the study, married participants were 1.2 times more likely to use contraceptives compared to their single counterparts and divorced participants were 9% less likely to use contraceptives compared to single participants. A study on family planning strategies across age categories^15^ reported that among women^15^^–24^, single women were more likely to use contraceptives compared to the married participants, this is in disagreement with the findings of our study. Another study on Modern contraceptive use among HIV-infected women attending HIV care centres in Togo reported that married women were less likely to use contraceptives. This is contract to our findings. It is important to note that participants of this study had an average age of 34.3 years and this could explain the variation of the findings16. Another factor that was significantly associated with acceptability of contraceptives among was having a friend that uses contraceptives. It has been reported that exposure to information on contraception use can influence use of contraceptives among young women17 and having a friend probably exposes one to information. These findings also agree a findings of a study on barriers and facilitators adolescent females living with HIV face on accessing contraceptives18, it was reported that peer influence was a key factor among adolescents that use contraceptives.

## Conclusion

There was a low contraceptives acceptability among young women living with HIV in Kampala city HIV clinics and this presents a challenge to a key component of the HIV control strategy among young women.

Contraceptives acceptability was associated with age, age at sex debut, having a friend that uses contraceptives and marital status.

## Study limitation

Collecting data from three facilities may have introduced clustering and this could have contributed to random error in the study, this was however minimized by adjusting for clustering in the analysis. The facilities in this study were purposively selected due to their high numbers of ART enrolment and this may have led to selection bias in the study, this was minimized by systematic sampling.

## Data Availability

Ethical restrictions have been imposed on the data in this study to protect the confidentiality of participant information. Interested researchers may submit queries related to data access to SOMREC (rresearch9@gmail.com) or corresponding author (wanixxl@gmail.com)

## References

[1] UNAIDS (2018). Global Factsheet 2018, HIV and AIDS Estimates.

[2] Uganda Bureau of Statistics Uganda demographic and health surveys.

[3] Ankunda Rachael, Muhimbuura Lynn, kiwanuka Noah (2016). sexual risk related behaviour among youth with HIV in central Uganda:implications for HIV prevention. The Pan African Medical Journal.

[4] Calvert C, Ronsmans C (2013). HIV and the risk of direct obstetric complications: a systematic review and metaanalysis. PLoS One.

[5] Vu L, Burnett-Zieman B, Banura C, Okal J, Elang M, Ampwera R (2017). Increasing Uptake of HIV, Sexually Transmitted Infection, and Family Planning Services, and Reducing HIV-Related Risk Behaviors Among Youth Living With HIV in Uganda. Journal of Adolescent Health.

[6] Magala I, Onega L, Rose N, Patrick S (2017). Factors influencing contraceptive uptake among sexually active HIV positive clients in TASO Masaka. Uganda J Public Health Policy Plan.

[7] Ministry of Health [Internet] (2016). Consolidated guidelines for prevention and treatment of HIV in Uganda, December 2016.

[8] Navajyoti Bora, Santosh kumar (2014). Acceptability and usage of contraceptive among women of reproductive age group in hilly areas of Garhwal, Uttrakhand, India. Journal of Dental and Medical Sciences.

[9] Campol Haynes Meagan, Ryan Nessa, Saleh Mona, Winkel Abigail Ford, Ades Veronica (2017). Contraceptive Knowledge Assessment: validity and reliability of a novel contraceptive research tool. Journal of Contraception.

[10] Whitaker AK, Dude AM, Neustadt A (2010). Correlates of use of long-acting reversible methods of contraception among adolescent and young adult women. journal of adolescent health.

[11] Mostafa Kamal SM (2012). Childbearing and the use of contraceptive methods among married adolescents in Bangladesh. The European Journal of Contraception & Reproductive Health Care.

[12] Hoopes AJ, Teal SB, Akers AY, Sheeder J (2018). Low Acceptability of Certain Contraceptive Methods among Young Women. J Pediatr Adolesc Gynecol.

[13] Samano R, Martinez-Rojano H, Chico-Barba G, Sanchez-Jimenez B, Sam-Soto S, Rodriguez-Ventura AL (2019). Sociodemographic Factors Associated with the Knowledge and Use of Birth Control Methods in Adolescents before and after Pregnancy. International Journal of Environmental Research and Public Health.

[14] Magnusson Brianna M, Masho Saba W, Lapane Kate L (2012). Early Age at First Intercourse and Subsequent Gaps in Contraceptive Use. Journal of Women's Health.

[15] Prata N, Bell S, Weidert K, Nieto-Andrade B, Carvalho A, Neves I (2016). Varying family planning strategies across age categories: differences in factors associated with current modern contraceptive use among youth and adult women in Luanda, Angola. Open Access Journal of Contraception.

[16] Yaya I, Patassi AA, Landoh DE, Bignandi EM, Kolani K, Namoro A-DD (2018). Modern contraceptive use among HIV-infected women attending HIV care centres in Togo: a cross-sectional study. BMJ Open.

[17] Gomez AM, Wapman M (2017). Under (implicit) pressure: young Black and Latina women's perceptions of contraceptive care. Contraception.

[18] Hagey Jill M, Akama Eliud, Ayieko James, Bukus Elizabeth A, Cohe Craig R, Pate Rena C (2015). Barriers and facilitators adolescent females living with HIV face in accessing contraceptive services:qualitative assessment of providers' perceptions in western Kenya. Journal of the International AIDS Society.

